# Direct-acting antiviral treatment in real world patients with hepatitis C not associated with psychiatric side effects: a prospective observational study

**DOI:** 10.1186/s12888-018-1735-6

**Published:** 2018-05-29

**Authors:** Isak Sundberg, Anders Lannergård, Mia Ramklint, Janet L. Cunningham

**Affiliations:** 1 Department of Neuroscience, Psychiatry, Uppsala University Hospital, Entrance 10, Floor 3B, 751 85 Uppsala, Sweden; 2 Department of Medical Sciences, Section of Infectious Diseases, Uppsala University Hospital, Entrance 34, Floor 2, 751 85 Uppsala, Sweden

**Keywords:** Hepatitis C virus, Direct-acting antiviral, Depression, Sleep, Side effects

## Abstract

**Background:**

Treatment of Hepatitis C virus (HCV) infection has evolved from interferon (IFN)-based treatments to direct-acting antivirals (DAAs). Patients with HCV have an elevated psychiatric morbidity (including substance abuse) and patients with such comorbidity have often been excluded from treatment with IFN. To date, little is known about psychiatric adverse effects of DAA-based regimens. We therefore aimed to study the psychiatric side effects of new IFN-free treatment for HCV (including depressive symptoms and sleep) in real world patients also including those with a history of psychiatric diagnosis, substance abuse or drug dependence.

**Methods:**

Consecutive patients were monitored during treatment with three of the latest DAA agents (sofosbuvir, simeprevir and daclatasvir). Repeated expert psychiatric assessments from baseline to 12 weeks post-treatment were performed with the Structured Clinical Interview for DSM-IV Axis I Disorders (SCID-I) clinical version and the self-report versions of the Montgomery Åsberg Depression Rating Scale (MADRS-S) and the Pittsburgh Sleep Quality Index (PSQI). Friedman’s test was performed to calculate differences in the MADRS-S and PSQI over time. In a post-hoc analysis Wilcoxon’s test was used to compare baseline depressive symptoms with those at post-treatment. Spearman’s rank correlation test was conducted in another post-hoc analysis to evaluate the correlation between symptoms of depression and HCV viral load at baseline.

**Results:**

At baseline, 15/17 patients (88%) had a history of any psychiatric diagnosis; 11 (65%) had a history of substance abuse or dependence; and 11 (65%) had previously been treated with IFN and six of those had experienced psychiatric side effects. There was no correlation between depressive symptoms and HCV viral load at baseline. Symptoms of depression did not increase during DAA treatment and were lower 12 weeks post-treatment compared with baseline: MADRS-S 10.7 vs. 8.3 (*p* = 0.01). This observation held when excluding patients taking antidepressant medication. Sleep quality did not significantly change during treatment. Adherence to treatment was estimated to 95% and sustained virological response was 88%.

**Conclusions:**

Despite high psychiatric morbidity, including previous substance abuse, patients successfully completed DAA treatment without increasing depressive symptoms or sleep disturbance. Symptoms of depression were significantly reduced 12 weeks after DAA treatment.

## Background

Hepatitis C virus (HCV) infection is an important cause of chronic liver disease worldwide with an estimated 185 million people infected. Moreover, it is among the leading causes of end-stage hepatic disease and is associated with the development of hepatocellular cancer [1]. Until 2011, the gold standard of care for HCV treatment was the combination of pegylated interferon alpha (PEG-IFN-α) and ribavirin (RBV), which are nonspecific immune boosters [2, 3]. A major disadvantage of this therapy has been frequent side effects that are largely attributed to IFN-α. Psychiatric side effects during IFN-α treatment include depressive symptoms in 30–70%, mild to moderate depression in 45–60% and major depression in 15–45% of treated individuals [4]. IFN-α triggers a series of hypothalamic-pituitary-adrenal axis abnormalities and immune responses, resulting in depressive symptoms [5]. Sleep disturbance is common in chronic HCV infection and treatment with IFN-α confers an additional risk of sleep disturbance of about 20% [6].

Psychiatric morbidity in patients with HCV infection is elevated and otherwise eligible patients have frequently not received treatment because of the fear of an exacerbation of psychiatric symptoms [4, 7–9]. A few studies suggest psychiatric comorbidity and drug abuse to be risk factors for non-adherence and not attaining sustained viral response (SVR) [10–12], whereas several other studies have demonstrated similar rates of adherence and SVR in patients with HCV infection and psychiatric comorbidity (including drug abuse) [13–17]. In a recent study the prevalence of drug use in the past year was 65% (201/309) in patients considering HCV treatment [18]. In the same material the prevalence of a lifetime psychiatric diagnosis was 88% and the prevalence of a current psychiatric diagnosis was 54% [19]. HCV infection per se may contribute to psychiatric symptoms by inflammatory routes, direct brain neurotoxicity, metabolic and neurotransmitter pathway derangement and immune-mediated responses [20, 21].

The arrival of direct-acting antiviral agents (DAAs) has drastically changed HCV treatment by increasing the likelihood of cure (referred to as SVR) and shortening the duration of treatment [22, 23]. The current generation of DAAs (e.g., daclatasvir [DCV], sofosbuvir [SOF], simeprevir [SIM] and ledipasvir [LDV]) is used without IFN [24, 25]. Because DAAs are not inflammatory cytokines, they should not share the same side effects as IFN-α and RBV of inducing flu-like symptoms, depression or suicidality. The side effect profile of DAAs compared with previous HCV medications is reported to be less severe [26] and patient-reported outcomes (PROs) improved [27, 28]. Although there are efficacy studies with DAAs that include patients with psychiatric comorbidity, [29, 30], few studies have specifically addressed psychiatric symptoms in DAA treatment [7, 13]. A recent retrospective study that excluded patients with substance abuse and prior IFN-based treatment found that symptoms of depression decreased after treatment with DAA. This study also reported equal adherence and SVR rates in patients with indications of mental disease compared with those without [31]. More data are needed to assess PROs and treatment adherence of patients with HCV in clinical practice [22, 28, 32].

Against this background, it is essential to study side effects, adherence and efficacy in a real-world patient population, where psychiatric comorbidity (including substance abuse) is common. Therefore, this study seeks to identify psychiatric side effects in HCV patients receiving DAAs (DCV, SOF and SIM), with repeated observations from baseline to 12 weeks post-treatment. To our knowledge, this is the first study to specifically monitor psychiatric side effects in DAA treatment in real-world patients with psychiatric morbidity including past substance abuse.

## Methods

### Subjects

This study was initiated in 2013, shortly before new DAA treatments were introduced, with the aim to study the longitudinal relationship between psychiatric symptoms and HCV treatment. Patients were recruited from a group of patients treated by a specialist in infectious disease (AL) at the Department of Infectious Diseases, Uppsala University Hospital. The eligible patients for DAA treatment were those without malignancy. Patients with advanced liver fibrosis and cirrhosis, extrahepatic diseases, as well as those with strong psychosocial reasons for treatment were prioritised for inclusion.

**Fig. 1:**
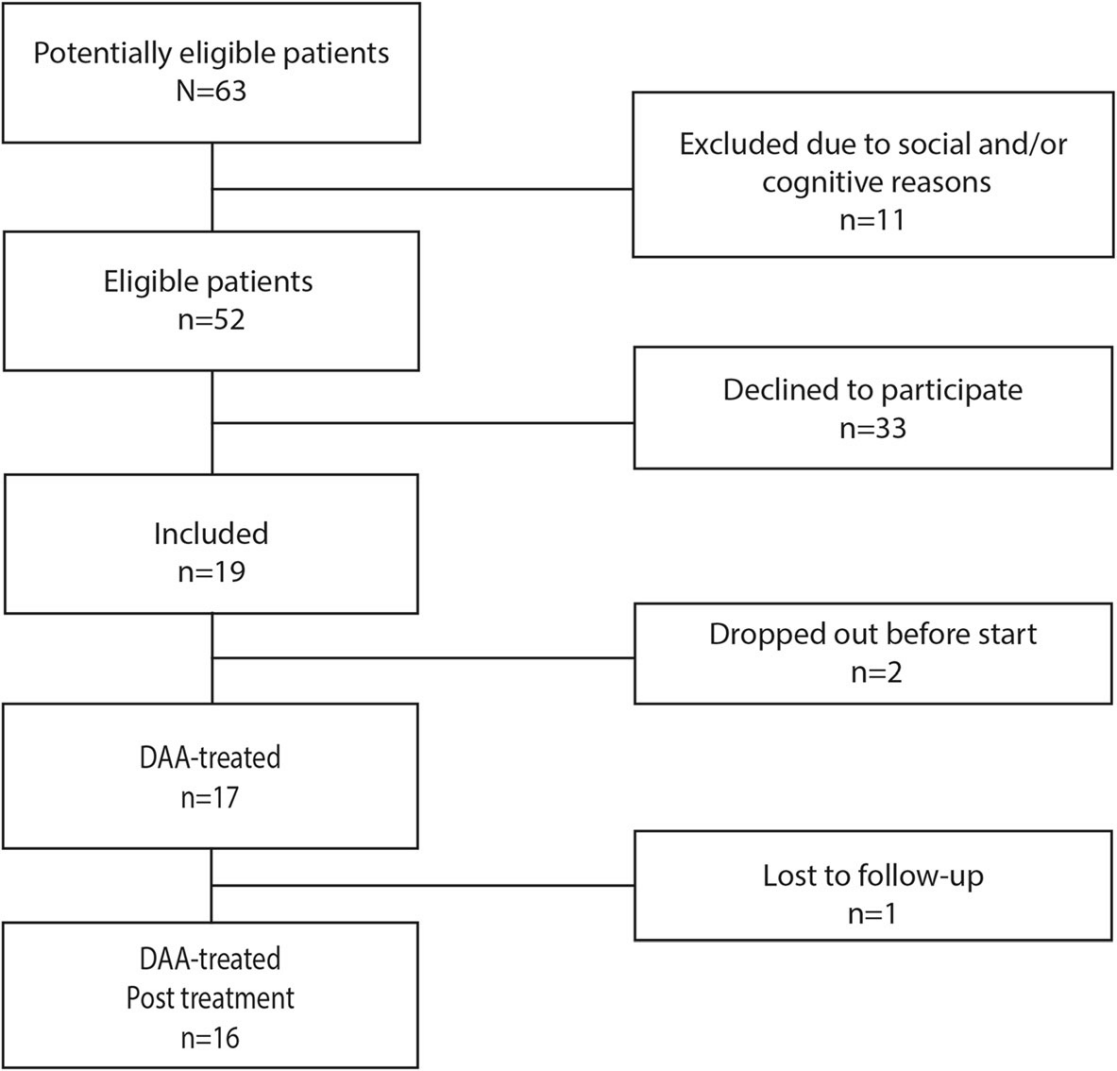
Patient inclusion. Sixty-three consecutive patients were considered for this study. Eleven were excluded because they could not speak or write fluently in Swedish or had low cognitive/intellectual ability. Of the 52 eligible patients, 19 accepted and were included in the study. Two patients declined to participate before study start. One patient was diagnosed with lung cancer and had to discontinue treatment at week 11. Sixteen of 17 patients completed the study

Exclusion criteria were inability to read or write Swedish and low cognitive or intellectual ability. These criteria were considered incompatible with completing an extensive research protocol and undergoing an in-depth inquiry with recurrent interviews. Sixty-three consecutive patients were considered for participation in this study between June 2014 and April 2015. Eleven were excluded because of a lack of Swedish language skills or low cognitive or intellectual functioning. This selection was made by the treating specialist (AL) at the Department of Infectious Diseases, Uppsala University Hospital, based on clinical judgement of the patient’s ability to understand and complete the study protocol with repeated assessments in the Swedish language. Of the 52 eligible patients, 19 (37%) accepted and were included in the study. Two of those patients declined to start the study before the first visit. Thus, the final sample was composed of 17/52 patients (33%). The patients represented the spectrum of individuals considered suitable for treatment of HCV. None of the patients had HBV-HCV or HIV-HCV co-infection. A flow chart of the patient inclusion process is provided in Fig. 1.

### Design

The study was a prospective observational study. The choice of treatment regimen was based on actual Swedish national recommendations by The Swedish Medical Products Agency at the time of treatment [33]. The prescription was combined with a treatment schedule that was carefully supervised by research nurses at the outpatient ward. Before treatment start, the physician reviewed laboratory data, liver elasticity, biopsy data and concomitant medication. A visit was scheduled every four weeks during treatment. After the treatment was completed, follow-up visits were performed at 12 and 24 weeks. At each time point, research nurses asked the participants about adherence to medication (taking DAA medication as prescribed or not), which was then noted in the medical records. Psychiatric assessment and patient self-report measures were employed at baseline, after 4 weeks of treatment, after 8 weeks of treatment, at the end of treatment and 12 weeks post-treatment.

Information was gathered on sociodemographic data, medical history and treatment from the patients and medical records. Patients and providers were blinded to the HCV RNA results at the time of completion of the questionnaires, but not blinded to the HCV RNA results from earlier time points in the study.

### Psychiatric assessment

At baseline, patients were assessed for past and current psychiatric morbidity by a trained psychiatrist (IS) using the Structured Clinical Interview for DSM-IV Axis I Disorders (SCID-I) clinical version. Previous HCV treatment and psychiatric side effects were also addressed in the interview. In following visits (including the 12-week post-treatment visit), patients were interviewed by the principal author, using module A of the SCID-I to assess the presence of depressive episodes according to DSM-IV.

### Self-assessment

Symptoms of depression were measured using the self-rating version of the Montgomery Åsberg Depression Rating Scale (MADRS-S). It has been shown to be a reliable and sensitive self-report tool for depressive symptoms [34] and thus suitable to follow patients with depressive symptoms over time. Scores on the MADRS-S range from 0 to 54, with higher scores indicating a greater severity of depression [35]. Sleep quality was measured with the Pittsburgh Sleep Quality Index (PSQI), a validated self-rated questionnaire assessing sleep quality and disturbances over a one month period [36]. The PSQI measures several aspects of sleep (e.g., sleep latency, sleep duration, habitual sleep efficiency and sleep disturbances). This tool includes seven component scores (range 0–3), as well as a composite global score (range 0–21). At each visit, patients filled in the MADRS-S and PSQI questionnaires. To screen for alcohol and drug use, the Alcohol Use Disorders Identification Test (AUDIT) [37] and Drug Use Disorders Identification Test (DUDIT) [38] were completed at baseline, after 12 weeks of treatment and 12 weeks post-treatment. The AUDIT is a 10-item screening instrument to assess alcohol consumption, drinking behavior and problems related to alcohol consumption. The DUDIT serves as a parallel tool to the AUDIT to identify persons with drug-related problems.

### Laboratory tests

HCV RNA was obtained from blood samples and analysed at each time point. All analyses were conducted at the Department of Clinical Chemistry at Uppsala University Hospital. A negative test for HCV RNA (detection limit of ˂15 IU/mL) at both 12 and 24 weeks post-treatment was considered as a SVR. The rate of the SVR was calculated on an “intention to treat” basis – i.e. all the patients who started treatment.

### Statistics

Non-parametric tests were used to calculate differences in MADRS-S, PSQI, AUDIT and DUDIT over time (Friedman’s test) and between depressive symptoms and HCV viral load at baseline (Spearman’s rank correlation test). In a post-hoc analysis Wilcoxon’s test was applied to compare baseline depressive symptoms with post-treatment depressive symptoms. All statistical analyses were conducted with the Statistical Package for the Social Sciences version 2 (SPSS Inc., Chicago, IL, USA). Reported *p*-values are two-sided with statistical significance set at *p* < 0.05.

## Results

Patient characteristics are presented in Table 1. All DAA-treated patients (17/17) received treatment with SOF combined with either SIM 9/17 (53%), DCV 7/17 (41%) or LDV 1/17 (6%). Additionally, 2/17 (12%) patients received RBV.

**Table 1:** Baseline characteristics and psychiatric morbidity

Age, mean (range)	58	(44–67)
Male sex, n (%)	9	(53)
BMI, mean (range)	27.2	(17.5–35.6)
HCV RNA IE/mL, median (range)	2.6 × 10^6^	(2.8 × 10^5^ - 16 × 10^6^)
HCV genotype, n (%)		
1a	7	(41)
1b	3	(18)
3a	5	(29)
1a/1b/1×	2	(12)
Descent		
European	16	(94)
Middle Eastern	1	(6)
Previous IFN treatment, n (%)	11	(65)
Psychiatric side effects during IFN treatment, n	6	
Liver cirrhosis, n (%)	10	(59)
Child-Pugh A	9	
Child-Pugh B	1	
Current psychiatric morbidity, n (%)	6	(35)
Any mood disorder	4	(24)
Any substance abuse or dependence^a^	2	(12)
Any anxiety disorder	1	(6)
ADHD^c^	2	(12)
Lifetime psychiatric morbidity, n (%)	15	(88)
Any mood disorder	10	(59)
Any psychotic disorder^b^	1	(6)
Any substance abuse or dependence	11	(65)
Any anxiety disorder	6	(35)
Any eating disorder	2	(12)
ADHD^c^	2	(12)
Psychotropic medication		
Any psychotropic medication	8	(47)
Antidepressants	3	(18)
Benzodiazepines	3	(18)
Benzodiazepine-like agents for sleep	3	(18)
Central nervous system stimulants	1	(6)
Buprenorphine	2	(12)

^a^ Patients were in an opiate substitution treatment programme

^b^ Substance-induced psychotic disorder

^c^ ADHD, attention-deficit/hyperactivity disorder based on patient diagnosis in medical records

### Treatment adherence and response

At 95% of the visits, patients reported taking their DAA medication as prescribed. Fifteen of 17 (88%) patients reached SVR at both 12 and 24 weeks. One male patient had a virological relapse. A female patient was diagnosed with lung cancer during the treatment; this individual had a negative HCV test (< 15 IU/mL) at 4 and 8 weeks of treatment and discontinued treatment at week 11.

One patient attained SVR but developed hepatocellular cancer shortly after completing DAA treatment. Ultrasound of the liver and computer tomography of the upper abdomen was performed before DAA treatment for this patient showing no indication of malignancy.

### Psychiatric morbidity at baseline

According to the SCID-I, 15/17 patients (88%) had any lifetime DSM IV psychiatric diagnosis. At baseline, 6/17 patients (35%) had an ongoing DSM IV psychiatric diagnosis. The number of lifetime psychiatric diagnoses ranged from 0 to 7 (median 2). Eleven of 17 patients (65%) had previous abuse or dependence of drugs or alcohol. Eleven of 17 patients (65%) were previously treated with IFN for HCV but the treatment had not been successful because of relapse (n = 3), intolerance (n = 3), partial response (n = 3) and viral breakthrough (n = 2). Retrospectively, at least two of the previously treated patients had developed a depressive episode during treatment (according to the psychiatric assessment with SCID-I) while another four had developed depressive symptoms. One patient had developed diabetes, which is a reported side effect of IFN/RBV treatment [39–41].

### Relationship between depressive symptoms and HCV viral load at baseline

There was no significant correlation between baseline symptoms of depression and HCV viral load at baseline (Spearman’s rho: *r* = 0.17, *p* = 0.55).

### Depressive symptoms during DAA treatment

**Fig. 2:**
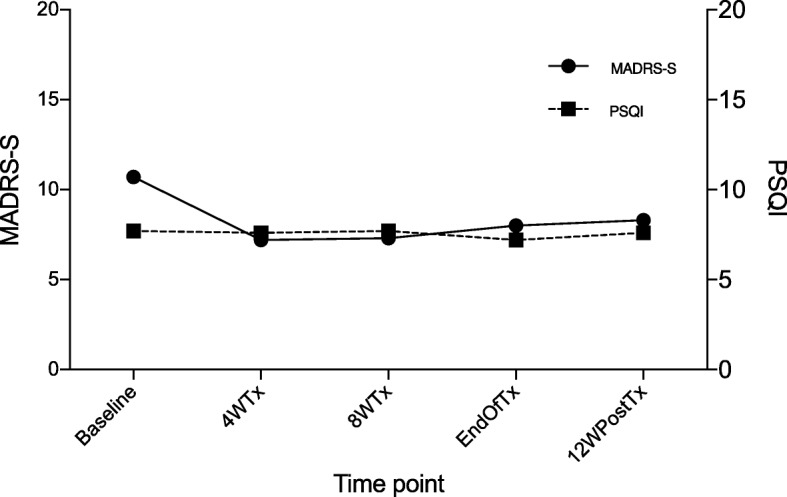
Mean scores for depressive symptoms (MADRS-S) and sleep quality (Total PSQI) from baseline to post-treatment. Depressive symptoms were lower 12 weeks post-treatment. Total PSQI did not significantly change during DAA treatment. Abbreviations: MADRS-S, self-rating version of the Montgomery Åsberg Depression Rating Scale; PSQI, Pittsburgh Sleep Quality Index; 4WTx, 4 weeks of treatment; 8WTx, 8 weeks of treatment; EOT, end of treatment; 12WpostTx, twelve weeks post-treatment

MADRS-S scores for each visit were available for 15/17 patients (one patient was lost to follow-up because of lung cancer and one missed filling in the MADRS-S at one of the assessment visits). For these 15 remaining patients, the mean MADRS-S score was 10.7 (range 1–30, *SD* 7.9) at baseline, 7.2 (range 0–26, *SD* 7.1) after 4 weeks, 7.3 (range 0–19, *SD* 5.7) after 8 weeks, 8.0 (range 0–25, *SD* 7.4) at the end of treatment and 8.3 (range 0–30, *SD* 8.3) at 12 weeks post-treatment (Fig. 2).

No statistical difference in MADRS-S levels was found when comparing all test points (Friedman test *Χ*^*2*^ = 4.03, *p* = 0.40), indicating that depressive symptoms did not increase during treatment. We observed a significant difference when comparing baseline MADRS-S with 12-week post-treatment (Wilcoxon *p* = 0.01), i.e. there were less depressive symptoms post-treatment.

Reanalysis, omitting those three patients taking antidepressant medication again found no statistical difference in MADRS-S levels when comparing all test points (Friedman test *Χ*^*2*^ = 4.6, *p* = 0.33) and a significant difference was still seen when comparing baseline MADRS-S with 12 weeks after treatment (Wilcoxon *p* = 0.02).

In one patient adjunctive RBV treatment was discontinued after four weeks because of affected blood count (from 146 to 115 g/L). For this patient, the MADRS-S score increased from 8 at baseline to 15 after four weeks and then decreased to 11, 9 and 8 during the rest of the study.

Three patients were depressed at baseline (based on the psychiatric assessment with the SCID-I). Two patients developed depression during DAA treatment. Of the two patients who developed depression, one described a subthreshold depressive episode at baseline (days with depressed mood but not during two weeks) and the other experienced family psychosocial problems during the treatment period. The latter patient also had a history of recurrent depressive episodes. One patient was depressed 12 weeks after treatment concluded.

### Sleep quality during DAA treatment

**Fig. 3:**
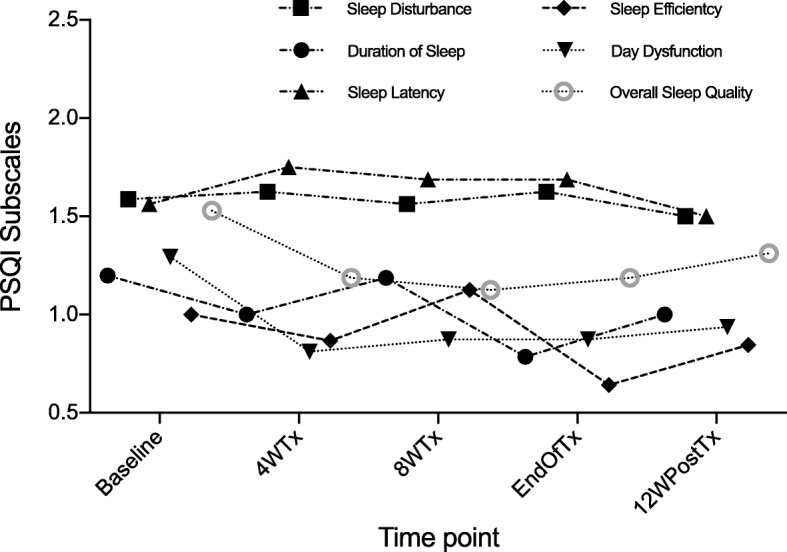
Mean component scores of the PSQI from baseline to post-treatment. There was no significant change in the component scores of the PSQI during DAA treatment. Abbreviations: PSQI, Pittsburgh Sleep Quality Index; 4WTx, four weeks of treatment; 8WTx, eight weeks of treatment; EOT, end of treatment; 12WpostTx, twelve weeks post-treatment

Mean total PSQI scores were 7.7 (range 2–19, *SD* 4.8) at baseline, 7.6 (range 2–18, *SD* 4.7) after 4 weeks, 7.7 (range 2–19, *SD* 4.9) after 8 weeks, 7.2 (range 2–19, *SD* 4.7) at the end of treatment and 7.6 (range 3–18, *SD* 4.9) 12 weeks after treatment ended (Fig. 2). No significant variation in total PSQI was noted (*Χ*^*2*^ = 3.4, *p* = 0.49). For PSQI component scores, post-hoc analysis of these measures revealed no significant variation during DAA treatment for sleep duration (*Χ*^*2*^ = 2.4, *p* = 0.66), sleep disturbances (*Χ*^*2*^ = 1.8, *p* = 0.77), sleep latency (*Χ*^*2*^ = 2.7, *p* = 0.61), day dysfunction (*Χ*^*2*^ = 5.5, *p* = 0.24), habitual sleep efficiency (*Χ*^*2*^ = 2.4, *p* = 0.67), and overall sleep quality (*Χ*^*2*^ = 3.4, *p* = 0.50) (Fig. 3).

### AUDIT during DAA treatment

Mean AUDIT scores were 1.9 (range 0–7, *SD* 2.2) at baseline, 1.5 (range 0–12, *SD* 3.0) 12 weeks of treatment and 1.9 (range 0–9, *SD* 2.5) 12 weeks post-treatment. There was no significant variation during treatment (*Χ*^*2*^ = 1.15, *p* = 0.59).

### DUDIT during DAA treatment

Mean DUDIT scores were 2.8 (range 0–41, *SD* 10.0) at baseline, 2.3 (range 0–28, *SD* 7.0) after 12 weeks of treatment and 0.6 (range 0–7, *SD* 2.0) 12 weeks after treatment. One person with previous methamphetamine abuse was taken into a substance abuse treatment centre just before start of the study (and start of treatment). This patient’s DUDIT scores were 41 at baseline, 28 after 12 weeks of treatment and 7 12 weeks post-treatment. She did not report drug abuse during the study, but because the questions in DUDIT refer to “during the last year”, high scores can be obtained without ongoing abuse. One patient had slightly increasing DUDÌT scores (0 at baseline, 4 after 12 weeks of treatment and 7 at 12 weeks post-treatment). No significant variation was found during the treatment (*Χ*^*2*^ *=* 1.2, *p* = 0.55).

## Discussion

To our knowledge, this prospective study is the first to specifically examine psychiatric symptoms during DAA treatment in real-world patients with substantial psychiatric morbidity (including a history of substance abuse). In this small but clinically relevant population of HCV patients with significant psychiatric comorbidity DAA treatment did not increase depressive symptoms or influence sleep quality. In line with previous results adherence in this group was high (> 95%) as was treatment response (88%) [26].

In this study, the MADRS-S score was significantly lower 12 weeks after the conclusion of treatment compared with baseline. This finding is in agreement with research showing that mental health parameters, neurocognitive function and fatigue are significantly improved [42–44]. Moreover, a reduction in Beck Depression Inventory scores was seen after treatment with SOF [31]. In addition to the psychological aspect of wellbeing in the virus-free patient [45], pretreatment viral load may contribute directly to inflammation in the central nervous system [20, 21, 45, 46] and to depressive symptoms. The eradication of HCV with anti-viral treatment may lower inflammation levels, resulting in less psychiatric symptoms [46–48]. In our sample, however, there was no correlation at baseline between viral load and depressive symptoms.

Two patients, who received RBV in addition to DAA, reported increased depressive symptoms during treatment (4–7-point increase in the MADRS-S score). There is no clear evidence linking RBV treatment to depressive symptoms [4]. However, previous studies have mainly focused on IFN-α. The depressive effects of IFN-α might have overshadowed smaller psychiatric side effects of RBV.

One strength of this study is its well-characterised patient sample. Another strength is that patients were assessed with gold standard psychiatric evaluation and followed prospectively for 9–12 months with repeated assessments, self-reported measures and clinical, biochemical and virological monitoring. The sample also reflects real world patients, which increases the generalisability of the findings. In contrast to many studies, our study includes patients with a history of substance abuse and IFN-based treatment.

One major limitation of this study is its small sample size. The relatively low inclusion rate was in part due to other ongoing studies at the clinic and may have produced a differential selection effect. A second limitation is that the assessment of language skills and cognitive functioning was based on subjective clinical judgement and was not operationalised formally. The results, such as the high adherence in this study, may therefore not be generalisable to populations with poor language skills and lower cognitive functioning. Yet, the study patients represent a selection of chronic HCV patients regarding psychiatric comorbidity and liver disease typical for our practice and with similar rates of psychiatric comorbidity as in other studies characterising psychiatric comorbidity in chronic HCV patients [18]. Patients’ use of psychotropic medication including antidepressants may have mitigated symptoms of depression and thus influenced outcome. This possibility is reflective of a real-life setting, however.

There is evidence that patients with intravenous substance abuse may be at a higher risk of reinfection, but that this risk can be lowered by properly addressing this comorbidity during and after HCV cure [49–51]. A coherent and comprehensive approach to deal with mental health and substance abuse is likely important to prevent reinfection of HCV.

## Conclusions

Although a majority of patients in this study had a history of affective disorder, drug abuse or neuropsychiatric disorder and previous IFN-based treatment, they were able to complete DAA treatment without substantial psychiatric side effects. Depressive symptoms were reduced after DAA treatment. Our study confirms findings showing that HCV patients with psychiatric comorbidity can be treated with DAAs with good efficacy and without psychiatric side effects, which is an important finding for HCV patients previously excluded from HCV treatment. Further studies with larger patient samples are needed to add strength to these findings.

## Abbreviations

AUDIT: Alcohol Use Disorders Identification Test
DAA: Direct-acting antiviral
DCV: daclatasvir
DUDIT: Drug Use Disorders Identification Test.
HCV: Hepatitis C virus
IFN-α: Interferon alpha
IU/mL: International units per milliliter
MADRS-S: The self-rating version of the Montgomery Åsberg Depression Rating Scale
PSQI: Pittsburgh Sleep Quality Index
RBV: Ribavirin
SIM: Simeprevir
SOF: Sofosbuvir
